# The Strategic Location of Glycogen and Lactate: From Body Energy Reserve to Brain Plasticity

**DOI:** 10.3389/fncel.2019.00082

**Published:** 2019-03-06

**Authors:** Corrado Calì, Arnaud Tauffenberger, Pierre Magistretti

**Affiliations:** Biological and Environmental Sciences and Engineering Division, King Abdullah University of Science and Technology, Thuwal, Saudi Arabia

**Keywords:** glycogen, lactate, astrocyte, ANLS, synaptic plasticity

## Abstract

Brain energy metabolism has been the object of intense research in recent years. Pioneering work has identified the different cell types involved in energy production and use. Recent evidence has demonstrated a key role of L-Lactate in brain energy metabolism, producing a paradigm-shift in our understanding of the neuronal energy metabolism. At the center of this shift, is the identification of a central role of astrocytes in neuroenergetics. Thanks to their morphological characteristics, they are poised to take up glucose from the circulation and deliver energy substrates to neurons. Astrocyte neuron lactate shuttle (ANLS) model, has shown that the main energy substrate that astrocytes deliver to neurons is L-Lactate, to sustain neuronal oxidative metabolism. L-Lactate can also be produced from glycogen, the storage form of glucose, which is exclusively localized in astrocytes. Inhibition of glycogen metabolism and the ensuing inhibition of L-Lactate production leads to cognitive dysfunction. Experimental evidence indicates that the role of lactate in cognitive function relates not only to its role as a metabolic substrate for neurons but also as a signaling molecule for synaptic plasticity. Interestingly, a similar metabolic uncoupling appears to exist in peripheral tissues plasma, whereby glucose provides L-Lactate as the substrate for cellular oxidative metabolism. In this perspective article, we review the known information on the distribution of glycogen and lactate within brain cells, and how this distribution relates to the energy regime of glial vs. neuronal cells.

## Introduction

L-Lactate was isolated in the 18th century and found to be released by muscle cells upon exertion, its physiological role been reduced, for a long time, to a simple waste product of anerobic metabolism. Interesting work in the ’80s started to unveil the metabolic properties of L-Lactate in skeletal muscles (Brooks, 1985). In contrast, our understanding of the energy metabolism in the central nervous system (CNS) was delayed because of the technical challenges in studying the brain compared to the peripheral organs. However, in the ’90s, it was proposed that astrocytes release L-Lactate as a result of aerobic glycolysis, i.e., the processing of glucose to lactate in the presence of physiological concentrations of oxygen, upon synaptic stimulation to support neuronal function, providing the first evidence of a lactate shuttle in the CNS (Pellerin and Magistretti, 1994; Magistretti and Allaman, 2018). This metabolic profile of astrocytes and their role in brain energy metabolism was initially received with skepticism, as mammalian cells are known to generate their main energy source molecule, ATP, within mitochondria, starting from glucose. Indeed, since glucose is almost fully oxidized by the brain, this implied that a transfer of L-Lactate from astrocytes to neurons should exist. ATP is mainly produced by oxidative phosphorylation, fueled by the tricarboxylic acid (TCA) cycle. Pyruvate originating from the glycolysis is transformed in a sequence of reactions to produce substrates supporting the TCA activity. Neurons are no different and express similar transporters for glucose (GLUT) at their membrane. Consistent with their high energetic demands neurons are mainly oxidative (80%–90% of their metabolism; Magistretti and Allaman, 2015). Questions then arise, as to why should neurons behave differently, and somehow rely on astrocyte-derived lactate to support their energy needs? Is this metabolic profile due to the specific expression of metabolic enzymes or because of the inability of neurons to store energy in the form of glycogen (Magistretti and Allaman, 2018)? Also, how do astrocytes sustain neuronal metabolism? In this short perspective article, we will briefly analyze these three points, and provide a review of recent evidence that address these questions.

## The Astrocyte Neuron Lactate Shuttle (ANLS)

According to the neuro-metabolism view up to the early ’90s, cells in the CNS simply need to consume glucose, whose constant supply needs to be provided by the vascular system, in order to sustain the brain homeostasis. Indeed, as already demonstrated by Sherrington at the end of the 19th century, blood flow is coupled to neuronal activity through a mechanism known as neurovascular coupling (Roy and Sherrington, 1890) mediated by a variety of vasoactive molecules (Magistretti and Allaman, 2015). This activity-dependent increase in local blood flow was considered sufficient to provide the necessary amount of glucose for the direct use by neurons. However, particularly upon intense neuronal activity, such as during long term potentiation (LTP), when synaptic plasticity requires additional energy support, glucose does not seem to be the preferred substrate to maintain neuronal activity (Suzuki et al., 2011). Experiments that investigated learning and memory formation in the context of the energy metabolism have shown that lactate, rather than glucose, was effective in reversing the amnestic effect caused by the inhibition of monocarboxylate transporters (MCTs) and of pharmacological inhibition of glycogenolysis, one of the mechanisms responsible for lactate production (Suzuki et al., 2011; Boury-Jamot et al., 2016; Gao et al., 2016). Supporting this view is the “Astrocyte-Neuron Lactate Shuttle (ANLS),” a neuroenergetic model first proposed in the ’90s, according to which glutamate uptake into astrocytes as a result of synaptic activity triggers an intracellular signaling cascade within astrocytes that results in the production of L-Lactate through aerobic glycolysis (Pellerin and Magistretti, 1994).

**Figure 1:**
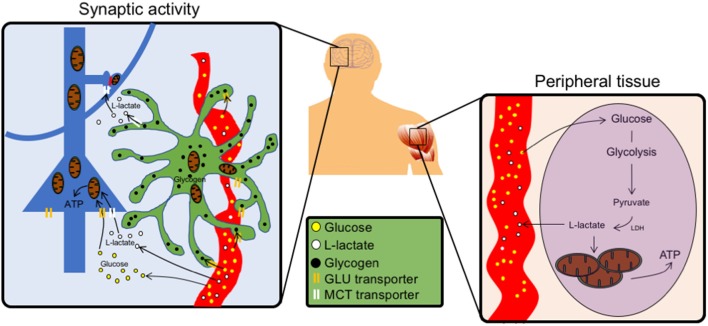
Schematics of the peripheral vs. central action of L-Lactate. **Left**, In the brain, neurons (blue) can uptake glucose using glucose transporters (GLUTs) and use it as a source of energy at the level of their soma. Around synapses, astrocytes (green) take up glucose from the blood vessels (red) and store it as glycogen granules (black). Upon synaptic activity, astrocytes produce L-Lactate in proximity of synapses, which express monocarboxylate transporter (MCT) transporters to import lactate as local source of energy. Lactate can also be formed from glycogen, the storage form of glucose. **Right**, In peripheral tissues glucose fuels tricarboxylic acid (TCA) cycle *via* circulating lactate through glycolysis.

The ANLS model reconciled, the morphological-based hypotheses of Camillo Golgi related to astrocytic metabolic support of neurons, with experimental evidence. *In vivo* experiments have demonstrated that indeed astrocytes are the predominant site of glucose uptake during synaptic activity (Figure 1). Thus, downregulating the expression of glutamate transporters on astrocytes drastically reduces the activity-dependent uptake of glucose into the brain parenchyma (Cholet et al., 2001; Voutsinos-Porche et al., 2003). A mirror experiment in which an increase in glutamate transporters in astrocytes was induced pharmacologically, resulted in an increase in glucose uptake into the brain parenchyma as determined by *in vivo* 2-deoxyglucose PET (Zimmer et al., 2017).

Additional *in vivo* experiments have shown that a gradient exists between the concentration of L-Lactate in neurons and astrocytes, favoring its efflux from astrocytes and its influx in neurons (Mächler et al., 2016), a phenomenon that has been also validated using computational models (Jolivet et al., 2015; Coggan et al., 2018).

Lactate is not only an energy substrate for neurons. Indeed, recent evidence, triggered by the observation that lactate transfer from astrocytes to neurons is necessary for LTP expression, synaptic plasticity and memory consolidation (Suzuki et al., 2011) has shown that lactate is also a signaling molecule for synaptic plasticity. Indeed lactate modulates the expression of at least 20 genes related to synaptic plasticity and neuroprotection (Yang et al., 2014; Margineanu et al., 2018). This signaling action of lactate is due to a positive modulation of N-Methyl-D-aspartate (NMDA) receptor signaling (Yang et al., 2014).

## Glycogen

Recent findings about the specific role of lactate derived from glycogen, rather than direct glycolysis of glucose, represents another modality through which the ANLS operates. Glycogen has a well-known structure, formed by linear chains of glucose that accumulates around a core protein called glycogenin, forming round granules of various size, between 20 and 80 nm in diameter in astrocytic processes (Calì et al., 2016). Glycogen was first discovered in peripheral tissues, and its concentration in the brain, compared to muscle and liver, is considerably lower, in a concentration ratio of 1:10:100, respectively (Magistretti and Allaman, 2013). Interestingly, glycogen granules are specifically located in astrocytes, although under pathological conditions they can accumulate in neurons, eventually to cause neurodegeneration, like in the Lafora disease (Magistretti and Allaman, 2007; Vilchez et al., 2007).

As glycogen is the storage form of glucose, it is safe to speculate about its physiological role as energy storage, which implies that astrocytes can be considered energy reservoirs. A pioneering work in the ’80s demonstrated how, in the cortex, two neuromodulators, vasoactive intestinal peptide (VIP) and noradrenaline (NA; Magistretti et al., 1981; Magistretti and Morrison, 1988), are potent glycogenolytic signals, a phenomenon resulting in local increase of phosphate-bound energy sources (ATP) within the stimulated networks (Magistretti and Schorderet, 1984). Recent evidence confirms these findings, expanding our understanding of the role of NA in particular, whose network activation is mobilized during attentional states necessary for cognitive functions such as learning and memory (Gao et al., 2016; Alberini et al., 2017). From a molecular point of view, NA binds to β2 adrenergic receptors in astrocytes, whose activation triggers glycogenolysis (Magistretti and Morrison, 1988; Sorg and Magistretti, 1992) and the subsequent rise of extracellular lactate levels that are needed for LTP and memory formation (Suzuki et al., 2011; Gao et al., 2016).

Therefore, given the role of lactate derived from astrocytic glycogen in synaptic plasticity, a functional relationship between astrocytic processes filled with glycogen and synaptic profiles is likely to exist (Calì et al., 2016, 2017; Agus et al., 2018). Indeed, recent reports using 3D electron microscopy have shown the preferential location of glycogen granules in astrocytic processes around synapses, rather than being randomly distributed in the astrocytic cytosol, both in the hippocampus and in the cortex (Calì et al., 2016; Mohammed et al., 2018). From an ultrastructural point of view, such distribution suggests that when high firing rate results in phenomena like LTP, that are translated into higher functions such as learning and memory stabilization, lactate, derived from glycogen stored within astrocytic granules close to synapses may exert its dual role of both energy substrate and signaling molecule for plasticity (Figure 1).

Sustained neuronal activity, like the one leading to LTP, does not merely induce a metabolic response in astrocytes, whose effect would be measurable after hours, but is also known to trigger an immediate calcium elevation (Araque et al., 2014; Bazargani and Attwell, 2016; Santello et al., 2019). Astrocytic calcium waves are diverse and complex (Di Castro et al., 2011; Agarwal et al., 2017; Bindocci et al., 2017), and their exact nature is still under debate, although evidence has shown their role in triggering glutamate release both *in vitro* and *in situ* (Bezzi et al., 1998). One potential mechanism involves exocytosis of synaptic-like microvesicles (Calì et al., 2008, 2009, 2014; Marchaland et al., 2008) upon activation of astrocytic GPCRs (Bezzi et al., 2004). It is worth mentioning that in a recent report, astrocytes have been shown to modulate levels of another monamine, dopamine, in the prefrontal cortex (Petrelli et al., 2018). These astrocytes express channels and enzymes that regulate homeostasis of dopamine, which could potentially modulate glycogen phosphorylase (GP) activity *via* cAMP (Smith et al., 2004). Furthermore, dopamine activation of D1-like receptors increases intracellular calcium levels, a mechanism likely to take place in astrocytes. Despite the link between LTP and calcium waves in astrocytes, a similar effect on metabolic substrates like lactate or glycogen is not known. A direct link has been reported between the activation of the store-activated calcium channels (SOCE) in astrocytes and glycogenolysis. This process serves as a glycolytic source of ATP to fuel SERCA pumps, to maintain adequate calcium levels in ER stores (Müller et al., 2014). Calcium, in particular, is an indirect signal for GP activation; therefore, one could speculate about the role of calcium in mobilizing energy stores in close proximity of microdomains. Conversely, calcium signaling in astrocytes might be affected by their metabolic turnover, as they depend on NAD+/NADH redox state, which is highly influenced by lactate fluxes (Requardt et al., 2012; Wilhelm and Hirrlinger, 2012).

## Peripheral Lactate Utilization

The role of L-Lactate is not limited to the CNS. Metabolism in peripheral tissues, and the action of lactate have also been extensively investigated in skeletal muscle, heart and in tumoral tissues. Several groups identified the presence of lactate dehydrogenase (LDH) on the mitochondria of sperm cells (Hochachka, 1980) and then in kidney, liver and muscle cells (Kline et al., 1986; Brandt et al., 1987). Brooks (1985) first named the cell-to-cell lactate shuttle in muscle, in 1985. Interestingly, the lactate shuttle is not limited to cytoplasm-mitochondria communication but also to cytosol-peroxisome where it supports the β-oxidation (McClelland et al., 2003). Lactate is produced continuously under aerobic conditions in skeletal muscle and oxidative muscle cells have the capacity to oxidize lactate present in the plasma or released by glycolytic muscle cells. It was also shown that rodent and human muscles cells possess the mitochondrial lactate oxidation complex (mLOC; Dubouchaud et al., 2000) that includes the presence of LDH isoforms, MCTs and cytochrome C oxidase, in their mitochondria. Furthermore, lactate can also be oxidized by mitochondria isolated from liver, heart and skeletal muscle cells.

During exercise, the oxidation of L-Lactate released by muscle cells increases up to 75%–80% of the basal values in the blood stream (Mazzeo et al., 1986) and it is now demonstrated that lactate can stimulate mitochondria biogenesis through activation of PGC1α (Hashimoto et al., 2007) which in turn influences the transcription of LDH isoforms to increase the ratio of LDHA/LDHB. This change promotes the formation of Lactate over pyruvate (Summermatter et al., 2013). In the heart, during exercise, it is also believed that lactate becomes the predominant source of energy compared to other metabolic sources (Gertz et al., 1988) and longitudinal studies have demonstrated that trained animals have reduced lactate blood levels, most likely due to an enhanced capacity to use it as a substrate by different organs (Bergman et al., 1999). Interestingly, it appears that during endurance exercise, significant amounts of brain derived neurotrophic factor (BDNF) are released in the bloodstream correlating with the release of L-Lactate (Schiffer et al., 2011). BDNF is an important trophic factor in the brain. L-Lactate has been demonstrated to increase BDNF expression in different neural cell systems (Coco et al., 2013; Yang et al., 2014). Interestingly, recent work has shown that cortical astrocytes can recycle BDNF and ultimately promote TrkB phosphorylation, to sustain LTP (Vignoli et al., 2016).

Besides its role in brain and muscle physiology, lactate has an important role in cancer cells. Tumors have high glycolytic metabolism even under normal O_2_ levels, a phenomenon known as the Warburg effect. This environment supports cancer cell survival and leads to accumulation of L-Lactate. This buildup has been reported to inhibit the migration of CD8+, CD4+ T-cells (Haas et al., 2015). Moreover, tumors producing high level of LDHA (favoring the conversion of Pyruvate to Lactate) have less positive outcomes (Brand et al., 2016). Blood lactate concentration observed around a tumorigenic environment can vary massively, raising from 1.5 to 3 mM in physiological conditions, up to 30–40 mM in cancerous tissues (Hirschhaeuser et al., 2011; Colegio et al., 2014; Haas et al., 2015). Moreover, the inhibition of the immune system by L-Lactate is not limited to a disturbance of immune cells metabolism, but also through an increase of pro-survival factor such as HIF-1α or angiogenic factors (Shi et al., 2011; Magistretti and Allaman, 2018).

Overall, lactate has been shown to be involved in multiple processes besides its metabolic support to muscle cells. L-Lactate ensures the survival of tumoral tissues by both promoting an environment favorable for their growth and reducing the reactivity of the adaptive immune system.

## Lactate as a Metabolic Buffer Between Glucose and Oxidative Metabolism in Peripheral Tissues

Recently a mechanism reminiscent of the ANLS has been shown to operate at the whole-body level (Figure 1). Indeed using *in vivo* Magnetic Resonance Spectroscopy to trace the fate of various metabolites in fed and starving mice, it was shown that ^13^C-lactate was extensively labeling TCA intermediates in peripheral organs (Hui et al., 2017). By measuring glucose metabolites in all organs, the authors found a considerably higher amount of circulating lactate compared to other metabolites, concluding that L-Lactate can act as a reservoir molecule whose turnover can be glycolytically fine-tuned on demand, rather than directly using glucose. This mechanism is reminiscent of what is observed in tumorigenic environment, where circulating lactate represents a more efficient way to use local energy reserves and uncouple it from glucose availability, which can be influenced by multiple factors. Interestingly, the only exception was the brain, where glucose was surpassing the amount of circulating lactate. As shown by the ANLS, a lactate gradient between astrocytes and neurons (Mächler et al., 2016) allows its exchange *via* the MCTs. Such a metabolic flow relies on astrocytic glycolysis, which is necessarily coupled to glucose utilization, triggered by synaptic signaling (Pellerin and Magistretti, 1994; Magistretti and Allaman, 2015). An even more tightly regulated way of energy delivery on demand occurs *via* lactate derived from glycogen (Suzuki et al., 2011; Gao et al., 2016). In this case, energy stores, under the form of glycogen granules, around synapses, can serve as metabolic reservoirs of lactate for energy delivery and plasticity signals for synapses. Consistent with the fact that astrocytes are locally synthesizing on demand lactate from glucose and glycogen, the amount of peripheral lactate accessing the brain is minimal (Hui et al., 2017).

From the above considerations, one should consider a dual action of lactate; one, as a source of energy, based on the uncoupling of glucose metabolism from the TCA cycle in astrocytes and the delivery of lactate to neurons. The second one, *via* the glycogen, as a signaling molecule for plasticity.

## Any Issues?

At its time of publication in 1994 (Pellerin and Magistretti, 1994), the ANLS model has been challenged, although the concept of the lactate shuttle, at least in the skeletal muscle, was not a novelty (Brooks, 1985). It is worth mentioning that most controversies around the ANLS raised from calculations inferred from theoretical models or metabolic stoichiometry rather than experimental data, opposing to the ANLS a model hypothesizing lactate flow from neurons to astrocytes, for disposal into the blood stream. (Dienel, 2012, 2017) To summarize, the few opponents to the ANLS argue that considering the rapid release of lactate in the bloodstream upon brain activity and the small concentration of lactate in the brain its oxidation in the neurons cannot support their synaptic activity. However, a compelling number of *in vivo* investigations have demonstrated that synaptic activity, and glutamate release trigger upstream intracellular cascades in astrocytes promoting glucose utilization mainly by astrocytes (Chuquet et al., 2010; Jakoby et al., 2014). Moreover, experimental work has also shown that upon glutamate activity, the glycolytic activity in the astrocytes is enhanced, compared to neurons (Mongeon et al., 2016) and that the loss of astrocytic glutamate transporters reduced the glucose consumption in activated brain areas (Cholet et al., 2001). Finally, some questions arose about the neuronal type that can support the model. For example, since GABA uptake by the astrocytes does not trigger aerobic glycolysis (Peng et al., 1994; Chatton et al., 2003) it is clear that energy delivery to GABA neurons operates through other mechanisms. However, since GABA neurons are mostly interneurons that are activated by glutamatergic inputs, it is conceivable that the glutamate-stimulated ANLS may provide energy to GABAergic neurons. Overall, converging evidence from several laboratories indicates that the ANLS provides an operational model for the coupling between neurons and astrocytes (Barros and Weber, 2018).

## Author Contributions

CC, AT and PM wrote the manuscript.

## Conflict of Interest Statement

The authors declare that the research was conducted in the absence of any commercial or financial relationships that could be construed as a potential conflict of interest.
